# *Campylobacter jejuni* enters gut epithelial cells and impairs intestinal barrier function through cleavage of occludin by serine protease HtrA

**DOI:** 10.1186/s13099-019-0283-z

**Published:** 2019-02-13

**Authors:** Aileen Harrer, Roland Bücker, Manja Boehm, Urszula Zarzecka, Nicole Tegtmeyer, Heinrich Sticht, Jörg D. Schulzke, Steffen Backert

**Affiliations:** 1 Division of Microbiology, Dept. of Biology, University of Erlangen-Nuremberg, Staudtstr. 5, 91058 Erlangen, Germany; 2 Institut für Klinische Physiologie, Med. Klinik m.S. Gastroenterologie, Infektiologie und Rheumatologie, Charité-Universitätsmedizin Berlin, Berlin, Germany; 3 Division of Bioinformatics, Institute of Biochemistry, Friedrich-Alexander-Universität Erlangen-Nürnberg, Erlangen, Germany; 4 Department of General and Medical Biochemistry, Faculty of Biology, University of Gdansk, 80-308 Gdansk, Poland

**Keywords:** Occludin, Tight junction, E-cadherin, *Campylobacter*, Protease, HtrA

## Abstract

*Campylobacter jejuni* secretes HtrA (high temperature requirement protein A), a serine protease that is involved in virulence. Here, we investigated the interaction of HtrA with the host protein occludin, a tight junction strand component. Immunofluorescence studies demonstrated that infection of polarized intestinal Caco-2 cells with *C. jejuni* strain 81–176 resulted in a redistribution of occludin away from the tight junctions into the cytoplasm, an effect that was also observed in human biopsies during acute campylobacteriosis. Occludin knockout Caco-2 cells were generated by CRISPR/Cas9 technology. Inactivation of this gene affected the polarization of the cells in monolayers and transepithelial electrical resistance (TER) was reduced, compared to wild-type Caco-2 cells. Although tight junctions were still being formed, occludin deficiency resulted in a slight decrease of the tight junction plaque protein ZO-1, which was redistributed off the tight junction into the lateral plasma membrane. Adherence of *C. jejuni* to Caco-2 cell monolayers was similar between the occludin knockout compared to wild-type cells, but invasion was enhanced, indicating that deletion of occludin allowed larger numbers of bacteria to pass the tight junctions and to reach basal membranes to target the fibronectin receptor followed by cell entry. Finally, we discovered that purified *C. jejuni* HtrA cleaves recombinant occludin in vitro to release a 37 kDa carboxy-terminal fragment. The same cleavage fragment was observed in Western blots upon infection of polarized Caco-2 cells with wild-type *C. jejuni*, but not with isogenic Δ*htrA* mutants. HtrA cleavage was mapped to the second extracellular loop of occludin, and a putative cleavage site was identified. In conclusion, HtrA functions as a secreted protease targeting the tight junctions, which enables the bacteria by cleaving occludin and subcellular redistribution of other tight junction proteins to transmigrate using a paracellular mechanism and subsequently invade epithelial cells.

## Introduction

*Campylobacter jejuni* are Gram-negative, motile bacteria with a spirally shaped body that commensally colonize the intestines of birds and mammals. However, in humans *C. jejuni* causes gastroenteric infections, and as such *C. jejuni* is among the most common causes of zoonotic illnesses worldwide. Infections are frequently caused by contaminated chicken meat and other animal-derived products. Infected individuals may sporadically develop secondary diseases such as Guillian–Barré or Miller–Fisher syndrome that are more serious than the usually self-limiting diarrhea in campylobacteriosis [1–3]. Upon reaching the gut, a first step in the pathogenic process leading to tissue damage is invasion of the bacteria into epithelial cells, as was demonstrated in biopsies of infected patients and by the use of in vitro infection assays [2, 4]. For this process, *C. jejuni* uses several outer membrane proteins to adhere to and invade into the cells, for instance CadF and FlpA, which bind to the extracellular matrix protein fibronectin followed by cell entry in an integrin-dependent fashion [5–9]. Interestingly, fibronectin and integrins are predominantly located on the basal side of enterocytes, but how *C. jejuni* reaches these basal receptors for a long time remained unknown. Paracellular transmigration of the pathogen is an intriguing possibility, and recently a protein that could be involved in this process was identified as the serine protease HtrA [10, 11].

Many bacteria contain one or more HtrA homologs [12–18]. HtrA proteins combine both protease and chaperone functions and are commonly located in the periplasmic space. Various HtrAs are composed of an amino-terminal signal peptide, a trypsin-like serine protease domain and one or two PDZ-domains responsible for protein–protein interaction [19, 20]. HtrA of *Escherichia coli* is the best studied model, and this species contains three homologs called DegP, DegQ and DegS. Their main function is to protect *E. coli* against heat and other stresses, and to remove misfolded proteins [19, 21, 22]. *C. jejuni* contains only one HtrA homolog, and this periplasmic protein can be secreted into the extracellular space, where it is able to cleave the extracellular domain of the adherens junction protein E-cadherin [10]. This helps *C. jejuni* to transmigrate between neighbouring cells to reach the basal side the polarized epithelium, a process that depends on HtrA activity [11, 23].

The question addressed here is how *C. jejuni* acts on tight junctions, which are located above the adherens junctions facing to the gut lumen and tighten the lateral intercellular space (LIS) to form a barrier against the intestinal lumen. Tight junctions are composed of a protein network localized at the apical site of epithelial and endothelial cell layers. Their so-called “fence” function maintains the cell’s polarity, while their “gate” function depends on openings, which only allow small molecules to pass the apical-basal barrier [24, 25]. Tight junction strands are formed by several proteins including tricellulin, occludin, claudins and junction adhesion molecules (JAMs) [25–27]. All these proteins interact with the tight junction plaque proteins like ZO-1, ZO-2 and ZO-3 or cingulin, which are linked to the intracellular actin cytoskeleton. The first strand-forming tight junction protein identified was occludin, which forms homodimers in the cellular membrane. It contains four transmembrane domains at the N-terminus forming two extracellular loops that participate in the tight junction and a long intracellular C-terminal tail. The first extracellular loop is rich in glycine and tyrosine residues [28], whereas the second loop contains two conserved cysteine residues that are important for homodimerisation [29]. The exact function of occludin is still unclear, but it has been suggested to regulate tight junction assembly and the distribution of other tight junction proteins within the tight junction strand meshwork [30–33].

The impact of *C. jejuni* on intestinal tight junctions is widely unknown, but it was shown that the bacteria can co-localize with occludin during infection of the E12 cell line [34]. Infection with *C. jejuni* alters the localization of occludin within the host cell membrane, leading to a re-distribution of the protein, but the involved molecular process remained unclear [35, 36]. In addition, treatment of T84 cells with enriched outer membrane vesicles (OMVs) derived from *C. jejuni* was associated with the cleavage of host cell proteins [37, 38]. Since HtrA can be incorporated as cargo into released OMVs or secreted as soluble protein, we investigated whether tight junction proteins are targeted by the protease activity of *C. jejuni* HtrA. The results presented here suggest that occludin is a direct cleavage target of *C. jejuni* HtrA, which results in opening of tight junctions followed by paracellular transmigration of the bacteria.

## Materials and methods

### *Campylobacter* strains

*Campylobacter jejuni* strain 81–176 was used in this study, together with an isogenic knockout mutant 81–176*ΔhtrA* and the complemented mutant 81–176*ΔhtrA/htrA* (Table 1), [11, 39]. All *C. jejuni* strains were grown on *Campylobacter* blood-free selective Agar Base containing *Campylobacter* growth supplement (Oxoid, Wesel, Germany) or on Mueller–Hinton (MH) agar plates amended with 30 μg/mL kanamycin or 20 μg/mL chloramphenicol at 37 °C under microaerobic conditions generated by CampyGen gas packs (Oxoid) for 48 h.

**Table 1:** Bacterial strains and plasmids

Strain/plasmid	Genotype	References
*C. jejuni* 81–176	Wild-type	[11]
*C. jejuni* 81–176 *ΔhtrA*	*C. jejuni* strain 81–176 with Δ*htrA* deletion, Cat^R^	[11]
*C. jejuni* 81–176 *ΔhtrA/htrA*	Δ*htrA* deletion complemented with wild-type *htrA* from *C. jejuni* 81–176, Kan^R^	[39]
*E. coli* BL21(DE3)pLysS	F–, *omp*T, *hsd*S_B_ (r_B_–, m_B_–), *dcm*, *gal*, λ(DE3), pLysS, Cat^R^	Promega
pKB1004	pET28a, wt *htrA* from the *C. jejuni* NCTC11168 strain, C-terminal 6× His tag, Kan^R^	[69]
pUZCj4	pET26b, wt *htrA* from the *C. jejuni* NCTC11168 strain, C-terminal 6× His tag, Kan^R^	This work

### Caco-2 cell cultures, infection assay and immunofluorescence staining

Caco-2 cells (ATCC HTB-37) were cultured in 6-well or 12-well plates with DMEM medium containing 4 mM glutamine (Invitrogen, Karlsruhe, Germany) and 10% FCS (Invitrogen). All *C. jejuni* strains grown on *Campylobacter* blood-free selective Agar Base plates were resuspended in BHI to desired optical densities (OD_600_ nm) followed by infection of Caco-2 cells with a multiplicity of infection (MOI) of 100. After infection for 24 h, the cells were washed with PBS and immunofluorescence staining was performed as described [40]. Briefly, cells were fixed with 4% paraformaldehyde (PFA) at room temperature for 10 min followed by permeabilization with 0.25% Triton-X100 for 1 min and blocking with 5% BSA in PBS for 1 h. Proteins were stained with α-occludin antibodies, recognizing the C-terminus of the protein (Thermo Fisher Scientific, Darmstadt, Germany, cat. nr. 42–2400) or α-*Campylobacter* antibodies (Dako, Glostrup, Denmark). Nuclei were stained using 4′-6-diamidino-2-phenylindole dihydrochloride (DAPI) (Thermo Fisher Scientific). As secondary antibodies FITC (fluorescein isothiocyanate)-conjugated goat α-rabbit and TRITC (tetramethylrhodamine isothiocynate)-conjugated goat α-rabbit (Thermo Fisher Scientific) were used. Samples were analysed using a Leica SP5 confocal fluorescence microscope and different lasers (Leica Microsystems, Wetzlar, Germany), performed at the OICE (FAU Erlangen-Nuremberg, Germany). Images were obtained via LAS AF computer software (Leica Microsystems) and optimized in brightness and contrast with ImageJ-win64 (version 2.0).

### Colon biopsies and confocal laser-scanning microscopy

Biopsies were obtained from patients who underwent routine colonoscopy due to acute diarrhea or for preventive examination of colon cancer. The biopsies were taken from the sigmoid colon as previously described [41]. The described symptoms were mainly watery diarrhea associated with abdominal cramps and stool frequencies of 10–20 times within 24 h. The diagnosis of infection by *C. jejuni* was approved by stool culture. The *C. jejuni*-positive patients were in the acute phase of infection between 3 and 7 days after the first symptoms appeared and revealed elevated C-reactive protein (CRP) levels ranging from 20 to 150 mg/L and boosted blood leukocyte counts of 11.7 × 10^9^/L to 16.7 × 10^9^/L [41]. The inflammatory parameters in the *C. jejuni*-negative control group were normal (data not shown). All individuals had been informed of the risks and gave written consent. The biopsies were directly fixed in Tissue-Tek (Sakura, Alphen a/d Rijn, The Netherlands) in liquid nitrogen, stored at − 80 °C and then cryo-sectioned for immunofluorescence staining. Primary antibodies recognizing human tight junction proteins, rabbit α-ZO-1 (# 61–7300) and mouse α-occludin (# 33–1500), were used for detection (Invitrogen, Carlsbad, CA, USA) as well as α-Campylobacter (Santa Cruz Biotechnology Inc., Santa Cruz, CA, USA, # sc-58101) at 1:100 dilutions. Secondary AlexaFluor antibodies from goat α-mouse or α-rabbit IgG (Invitrogen) were used at 1:500 dilution. The tight junction proteins ZO-1 and occludin were visualized by confocal laser-scanning microscopy (Zeiss LSM510, Jena, Germany) and analyzed by Zeiss Image Examiner software.

### Inactivation of occludin in Caco-2 cells by CRISPR/Cas9

The occludin gene was inactivated in Caco-2 cells by application of the CRISPR/Cas9 technology. For this purpose, two plasmids were obtained from Santa Cruz (Heidelberg, Germany), one of which contained the 20 nucleotide gRNA for a region in the occludin gene [Occludin CRISPR/Cas9 KO Plasmid (human), sc-418271] and the HDR-plasmid [Occludin HDR-Plasmid (human), sc-418271-HDR] that was used for stable transfection. The procedure of mutagenesis was performed using a protocol provided by the manufacturer.

### Cloning, expression and purification of HtrA *C. jejuni*

The *htrA* gene of *C. jejuni* strain 11168 was introduced into *Nco*I and *Xho*I restriction sites of the pET26b expression plasmid, giving rise to vector pUZCj4. The *E. coli* BL21(DE3)pLysS strain transformed with pUZCj4 was used to overproduce wild-type HtrA with the C-terminal 6× His tags of the pET System (Novagen, San Diego, CA, USA). The bacteria were grown at 37 °C in Luria–Bertani (LB) broth supplemented with kanamycin (50 µg/mL) to OD of 0.8. Next, the HtrA expression was induced by addition of 0.5 mM isopropyl-β-d-thiogalactopyranoside (IPTG). After induction overnight, the temperature of the bacterial culture was reduced to 30 °C. The bacteria were centrifuged (10 min, 5000×*g*) and the pellet was resuspended in 15 mL of the lysis buffer BH10 (50 mM HEPES pH 8.0, 300 mM KCl, 10 mM imidazole pH 8.0). Afterwards, the cells were lysed by addition 1 mg/mL lysozyme followed by sonication. Next, to remove DNA from lysates, DNase I (5 µg/mL) was used. Lysates were cleared by centrifugation 25,000×*g* for 30 min at 4 °C. The nickel-affinity chromatography (Ni–NTA, Qiagen, Germany) under native conditions was applied to purify the protein as described by Zarzecka and co-workers [42]. Briefly, non-specifically bound proteins were washed from the loaded resin with buffer BH40 (50 mM HEPES pH 8.0, 300 mM KCl, 40 mM imidazole pH 8.0), and HtrA was eluted with buffer BH200 (50 mM HEPES pH 8.0, 300 mM KCl, 200 mM imidazole pH 8.0). The purity of the proteins was estimated to be more than 95% as judged by SDS-PAGE electrophoresis.

### Immunoblotting

Proteins were resolved in 12% SDS–PAGE gels and blotted as described [43]. Prior to antibody incubation, the membranes were blocked in TBST buffer (140 mM NaCl, 25 mM Tris–HCl pH 7.4, 0.1% Tween-20) with 3% BSA or 5% skim milk for 1 h at room temperature. The first antibody, rabbit α-occludin (H-279, sc-5562), mouse α-occludin (E5) (sc-133256, Santa Cruz), mouse α-β-Actin (Sigma Aldrich, Taufkrichen, Germany), or rabbit α-HtrA (courtesy of Lone Brøndsted, [44]) as indicated, was incubated overnight at 4 °C. As secondary antibody, horseradish peroxidase-conjugated α-rabbit or polyvalent α-mouse immunoglobulin was used (Life Technologies, Darmstadt, Germany). Antibody detection was performed with the ECL Plus chemiluminescence Western Blot kit (GE Healthcare Life Sciences, Munich, Germany) [45].

### Transwell assay

Caco-2 cells or the corresponding occludin knockout cells were grown for 14 days to confluent monolayers in a transwell filter system as described and the transepithelial electrical resistance (TER) was determined every 2 days [11, 39].

### Gentamicin protection assay

Caco-2 cells and the occludin knockout cells were infected with *C. jejuni* 81–176, Δ*htrA* and Δ*htrA/htrA* at an MOI of 100. After infection for 6 h, the cells were washed three times with 1 mL of pre-warmed DMEM medium per well to remove non-adherent bacteria. To determine the colony forming units (CFU) corresponding to intracellular bacteria only, extracellular bacteria were killed by incubation with 250 μg/mL gentamicin (Sigma Aldrich) at 37 °C for 2 h, after which the cells were washed three times with medium [46]. Cells were then lysed with 1 mL of 0.1% (w/v) saponin (Sigma Aldrich) in PBS at 37 °C for 15 min and resuspended, diluted, and plated on MH agar plates. To determine the total CFU corresponding to cell-associated bacteria (intra- and extracellular combined), following the three wash steps the infected monolayers were directly incubated with 1 mL of 0.1% (w/v) saponin in PBS at 37 °C for 15 min without prior treatment with gentamicin [47]. All experiments were performed in triplicates.

### In vitro cleavage of recombinant occludin

Three µg of recombinant GST-tagged human occludin (antikoerper-online.de) was incubated with 1 µg of purified *C. jejuni* HtrA in 25 µL HEPES buffer (50 mM, pH 7.4) for 16 h at 37 °C. To remove LPS, aliquots of 200 μg/mL HtrA were incubated with 20 μg/mL of polymyxin B (Sigma Aldrich) for 1 h at room temperature as described [48] and did not change the activity of HtrA and outcome of the experiments. The resulting cleavage products were analysed by immunoblotting stained with either α-occludin H-279 or with rabbit α-GST antibodies (GE Healthcare, Freiburg, Germany).

### Bioinformatics

The secondary structure in the vicinity of the cleavage site was assessed using PSIPRED [49]. Potential HtrA cleavage sites were detected by a pattern-based search using PATTINPROT (https://npsa-prabi.ibcp.fr/cgi-bin/npsa_automat.pl?page=/NPSA/npsa_pattinprot.html). The search pattern [VITA]-[VITA]-x(2,4)-[DN] was derived from an inspection of *H. pylori* HtrA cleavage sites in E-cadherin [50].

### Statistics

All data were evaluated via two-tailed Mann–Whitney test with GraphPad Prism 6 (Version 6.01). The obtained p-values *p *≤ *0.05* (*), *p *< *0.01* (**), *p *< *0.0001* (****) were defined as statistically significant.

## Results

### Redistribution of occludin in Caco-2 cells during *C. jejuni* infection depends on HtrA

**Fig. 1:**
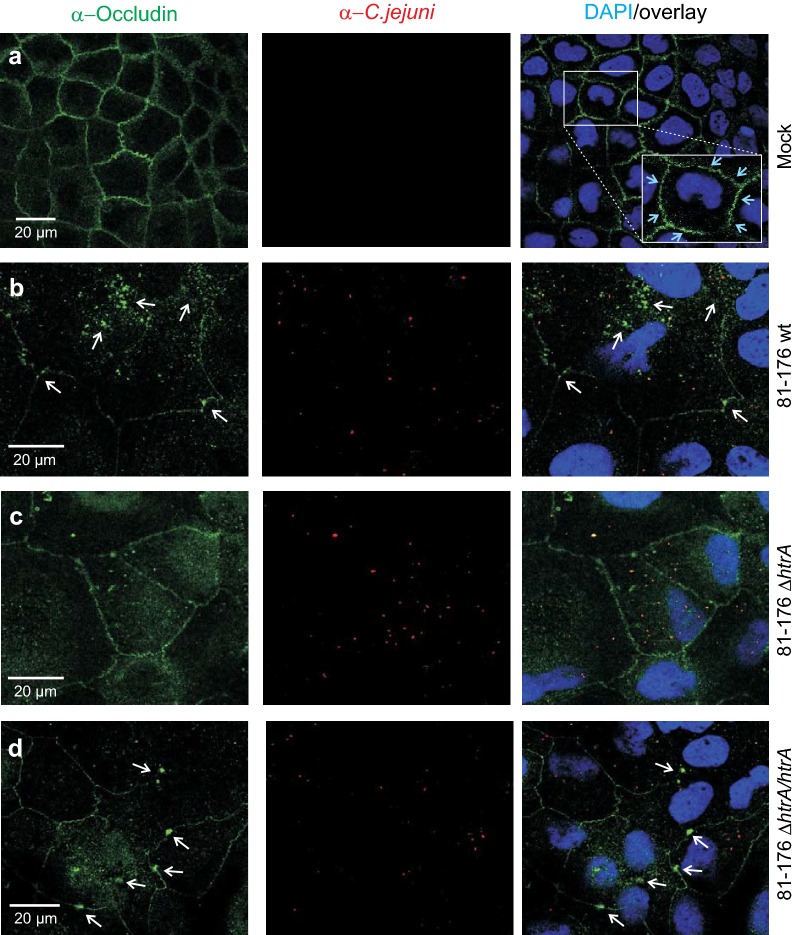
Infection of Caco-2 cells with *C. jejuni* disturbs the occludin patterns in a *htrA*-dependent manner. α**-**Occludin (green) and α-*C. jejuni* immunostaining of Caco-2 mock control cells (**a**) or cells infected with wild-type (wt) strain 81–176 (**b**), Δ*htrA* knockout mutant (**c**) or Δ*htrA* complemented with wild-type *htrA* (**d**). The inlay in panel A shows the belt-like pattern of occludin around the polarized cells as expected (blue arrows). Infection was performed for 12 h at an MOI of 100. The pictures revealed a redistribution of upon wt infection but not Δ*htrA* mutant (white arrows). Co-localization of occludin with *C. jejuni* was not observed. DAPI staining (blue) was used for visualization of the DNA in the nuclei

The effect of *C. jejuni* HtrA on the localization of occludin in the tight junctions was studied by immunofluorescence staining of in vitro infected cells. Confluent grown monolayer of polarized Caco-2 cells were infected with wild-type strain 81–176, its isogenic Δ*htrA* knockout mutant, or Δ*htrA* complemented with wild-type *htrA* (Δ*htrA/htrA*) as control. After infection for 24 h, the cells were fixed and stained for immunofluorescence visualization of occludin (green) and *C. jejuni* (red). This demonstrated that proper tight junctions with belt-like pattern of occludin are formed in the non-infected control (Fig. 1a, inlay, blue arrows), while infection with wild-type or complemented *C. jejuni* interrupted the typical occludin distribution (Fig. 1b, d, white arrows). Interestingly, this effect was not seen when the *htrA* gene was deleted (Fig. 1c). Thus, infection of polarized Caco-2 cells with bacteria containing an intact *htrA* gene altered the localization of occludin (Fig. 1b, d). These observations demonstrate that HtrA is involved in the disturbance and relocalization of occludin during infection. However, co-localization of occludin with the bacteria was not observed.

### Tight junction protein distribution in biopsies from campylobacteriosis patients

**Fig. 2:**
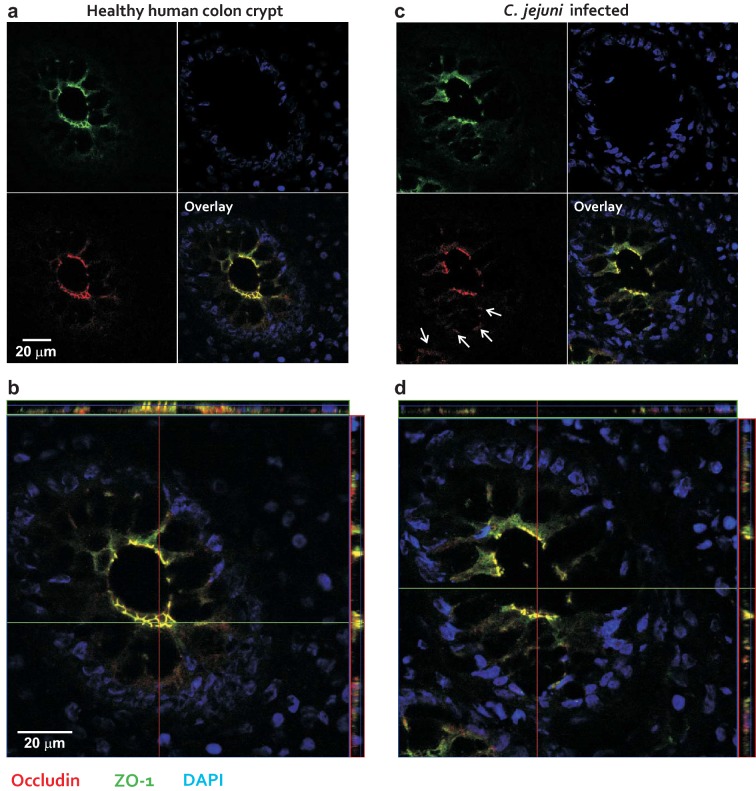
Tight junction protein distribution in intestinal biopsies from healthy individuals and campylobacteriosis cases. Confocal laser-scanning microscopy of human colon crypts from a non-infected individual (**a**, **b**) and an acute *C. jejuni* infection (**c**, **d**). The images show clockwise immunostaining for zonula occludens protein-1 (ZO-1, green), occludin (red), nuclei by use of DAPI staining (blue) and a merged image in which co-localization of the tight junction proteins ZO-1 and occludin appears as yellow. White arrows indicate redistributed occludin signals in the infected biopsies

**Fig. 3:**
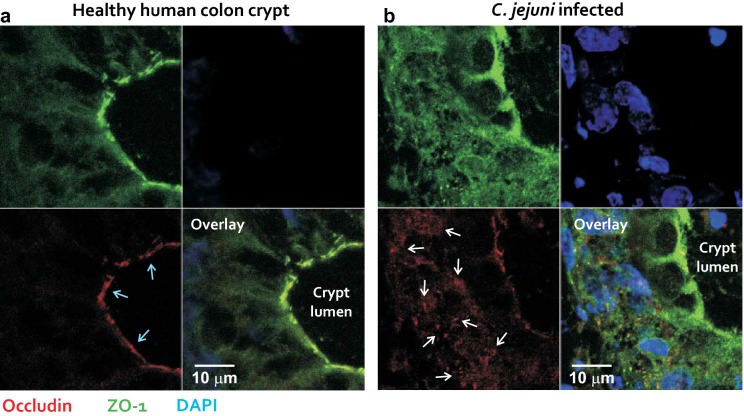
Enlarged images of gut epithelial cells from biopsies of non-infected and *C. jejuni*-infected patients. Confocal laser-scanning microscopy images at high magnification show in detail colon crypts from a healthy patient (**a**) and patient infected with *C. jejuni* (**b**). The biopsies are stained against occludin (red), ZO-1 (green) and the DNA with DAPI (blue). The overlay images (bottom, right) show the co-localization from occludin and ZO-1 (yellow) in the non-infected control (**a**) and in a disturbed tight junction pattern in the infected sample (**b**). Blue arrows indicate the apical labelling of occludin in non-infected controls, while white arrows indicate cytosolic occludin signals in the infected samples

To confirm that the in vitro observations had relevance to *C. jejuni* infection of humans in vivo, the subcellular distribution of tight junction proteins was investigated by confocal laser-scanning microscopy of three human colon biopsies obtained from acutely *C. jejuni* infected patients and from healthy controls, respectively. The absence or presence of *C. jejuni* in all samples was confirmed by antibody staining as well as stool culture as described [41]. In biopsies from non-infected controls, overview images showed that occludin was co-localizing with zonula occludens protein-1 (ZO-1) within the tight junctions as expected (Fig. 2a, b). In contrast, mucosal biopsies obtained from patients infected with *C. jejuni* revealed the redistribution of occludin and ZO-1 (Fig. 2c, d, white arrows). Close inspection of enlarged sections showed that occludin and ZO-1 changed their localization from predominantly apical sites in the controls (Fig. 3a, blue arrows) and became visible intracellularly after infection (Fig. 3b, white arrows). These data confirm that occludin and also other tight junction proteins like ZO-1 are relocated during acute *C. jejuni* infection.

### Knockout of occludin in Caco-2 cells weakens the tight junctions

**Fig. 4:**
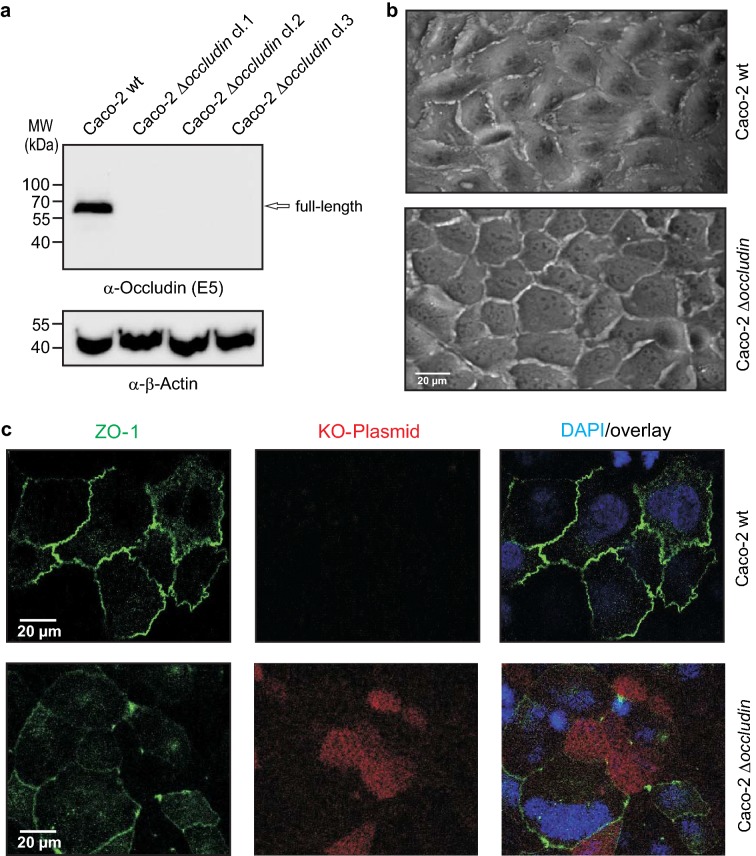
CRISPR/Cas9 knockout of occludin in Caco-2 cells reveals partial downregulation of ZO-1. Three individual occludin knockout clones of Caco-2 cells were analysed. Western blotting confirms the presence of occludin in Caco-2 wild-type (wt) cells and absence in the knockout cells (**a**). Phase contrast microscopy of Caco-2 wt cells (top) and occludin knockout cells (bottom) revealed only slight phenotypic differences (**b**). Immunofluorescence staining of Caco-2 wt (top) and occludin knockout cells (bottom) stained for ZO-1 (green), presence of the HDR-plasmid (red) and DNA in the nuclei by DAPI (blue). The red fluorescence derives from the CRISPR–Cas9 plasmid, which was used for stable transfection (**c**)

**Fig. 5:**
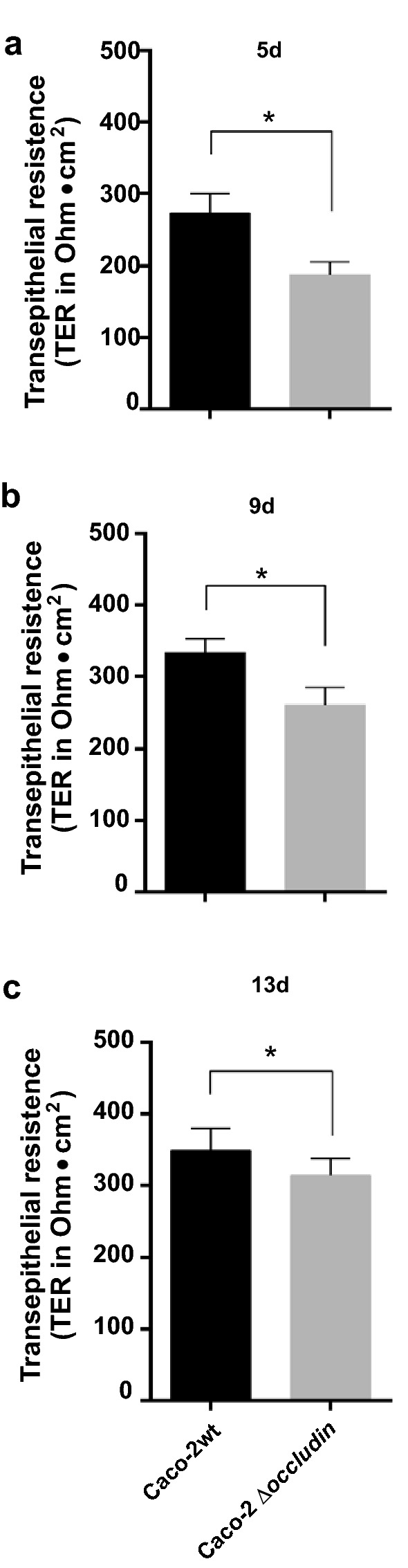
Transepithelial resistance (TER) in Caco-2 wild-type (wt) and occludin knockout during cell differentiation monitored over 2 weeks. The TER measurements revealed increasing TER values both in the wt and knockout Caco-2 cells over 5 days (**a**), 9 days (**b**) and 13 days (**c**). However, the values in the occludin knockout cells were always slightly below wt levels, indicating a significant permeability defect in the knockout. n = 15*, p *< 0.05 (*), Mann–Whitney test. Data represented mean ± SEM

In order to investigate the role of occludin relocalization by *C. jejuni* further, we inactivated the corresponding gene in Caco-2 cells by CRISPR/Cas9 mutagenesis. Absence of occludin expression in three knockout cell clones was confirmed by Western blotting using a monoclonal α-occludin antibody (Fig. 4a). The α-β-actin control blot demonstrated that equal protein was loaded on the gel. Phase contrast microscopy revealed only slight phenotypical differences between the confluent cell monolayers (Fig. 4b). To assess whether inactivation of occludin in Caco-2 cells introduced any changes in the tight junctions, the cells were stained for ZO-1 protein. This identified that inactivation of occludin caused a slight decrease of ZO-1 assembly in tight junctions, while the amount of ZO-1 in the cytoplasm increased (Fig. 4c). In addition, the functionality of the tight junctions was investigated by determining the transepithelial electrical resistance (TER). For this purpose, Caco-2 wild-type and occludin knockout mutant cells were grown for 13 days in a Transwell filter system to reach a confluent monolayer, and TER was measured every 2 days. The overall TER values increased during this period, however, significantly lower values were obtained for the knockout compared to wild-type cells (Fig. 5a–c). Thus, inactivation of occludin in Caco-2 cells leads to a slight destabilization, but not complete disruption of the tight junctions.

### Adhesion and invasion of *C. jejuni* depends on expression of occludin

**Fig. 6:**
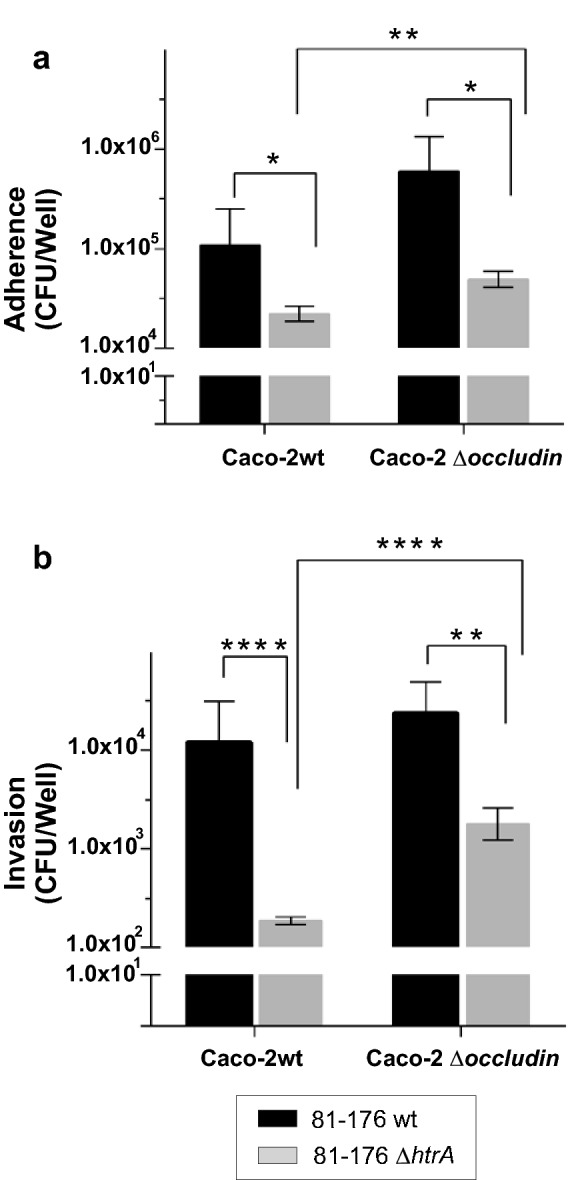
Quantification of cell-associated (adhesion) and intracellular (invasion) levels of *C. jejuni* infecting Caco-2 wild-type (wt) and occludin knockout cells. The two cell lines were infected for 12 h with the indicated strains using an MOI of 100. The total number of cell-associated (**a**) and intracellular bacteria (**b**) was determined by gentamycin protection assay. The adhesion and invasion levels were dependent on the cell type and expression of the *htrA* gene. n = 9*, p *< 0.05 (*), *p *< 0.01 (**), *p *< 0.0001 (****), Mann–Whitney test. Data represented mean ± SEM

Next, adhesion and invasion of *C. jejuni* upon infection of confluent wild-type Caco-2 and occludin knockout cells was determined by the gentamicin protection assay (Fig. 6). We observed that the Δ*htrA* deletion mutant adhered to and invaded into either cell line at significant lower levels compared to wild-type bacteria (Fig. 6a, b). In addition, the binding and invasion capacity of each strain was slightly higher for the occludin knockout cells compared to wild-type Caco-2, and reached statistical significance for the Δ*htrA* mutant. These results suggest that disturbance of occludin in polarized Caco-2 cells can enhance the adhesion and invasion capabilities of *C. jejuni*, probably as a result of weaker and more penetrable tight junctions.

### HtrA cleaves occludin upon *C. jejuni* infection of Caco-2 cells

**Fig. 7:**
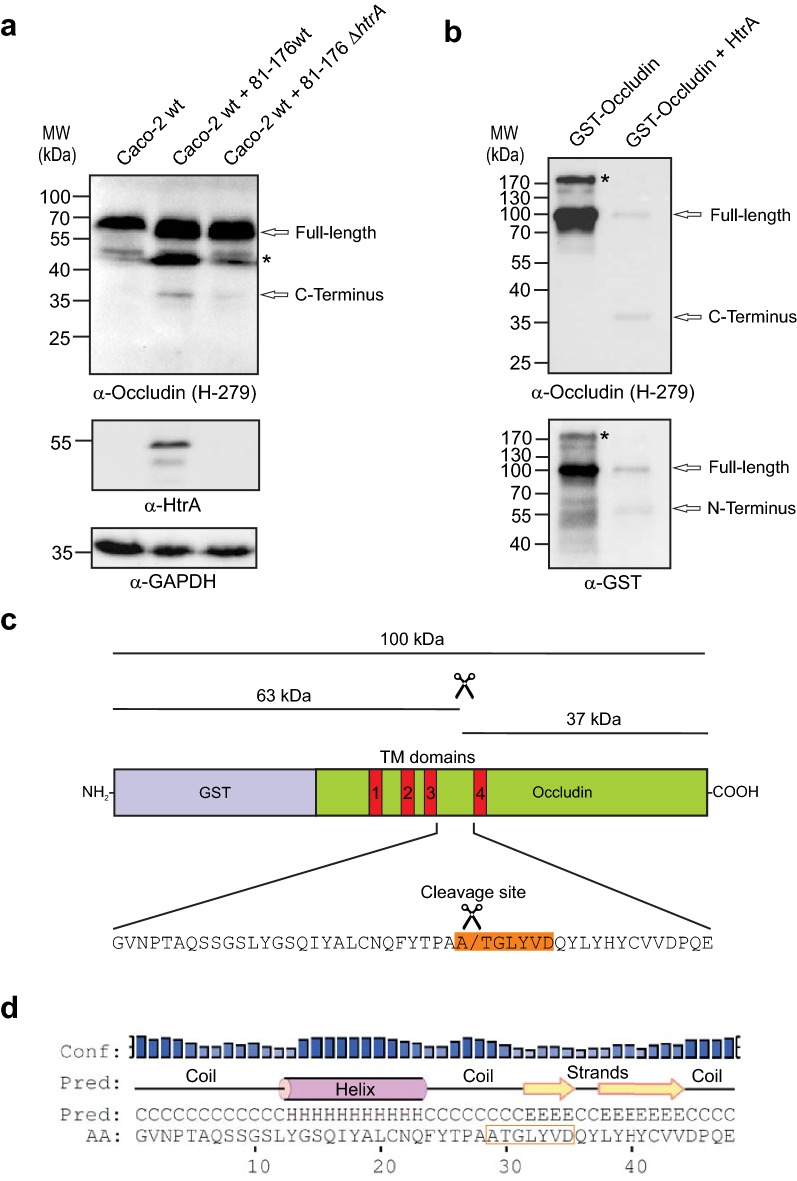
HtrA cleaves occludin during *C. jejuni* infection in vivo and in cleavage assays in vitro. **a** Immunoblotting of protein extracts from polarized Caco-2 cell monolayers infected with *C. jejuni* wild-type (wt) or *ΔhtrA* mutant. The blots were stained with polyclonal α-occludin antibodies recognizing the C-terminus of the protein. The α-HtrA and α-GAPDH blots served as controls. Besides the full-length protein (65 kDa), a cleaved C-terminal fragment (37 kDa) was visualized with α-occludin antibodies upon infection with wt bacteria. The asterisk marks a double band at ~ 45–50 kDa, presumably corresponding to two isoforms of occludin recognized by this antibody. **b** Recombinant GST-tagged human occludin was incubated with purified HtrA in in vitro cleavage assays. Cleavage of full-length GST-occludin (monomer at 100 kDa) resulted in the production of the same 37 kDa C-terminal fragment as detected upon infection. Reprobing of the blot with α-GST antibodies resulted in the detection of the corresponding N-terminal cleavage fragment of 63 kDa. The asterisks mark the position of GST-occludin dimers migrating at ~ 200 kDa. **c** Mapping of the occludin cleavage fragments. An HtrA cleavage site was localized in the second extracellular loop between the transmembrane (TM) domains 3 and 4 as indicated. Bioinformatical investigation revealed the exact cleavage position as indicated on the bottom. **d** Secondary structure prediction for the second extracellular loop. The predicted type of secondary structure (Helix ‘H’, Coil ‘C’, and Strand ‘E’) is shown above the sequence. The top line gives the confidence of the predictions, with high bars indicating a more reliable prediction

The above results suggest that *C. jejuni* HtrA has an impact on the function of occludin. To test if *C. jejuni* HtrA is involved in cleaving occludin, confluent Caco-2 monolayers were infected with wild-type and Δ*htrA* mutant *C. jejuni* for 24 h followed by immunoblotting using the polyclonal α-occludin (H-279) as well as α-HtrA and α-GAPDH antibodies as controls (Fig. 7a). The results show that besides a band of full-length occludin at 65 kDa and two isoforms (~ 45–50 kDa, asterisk), an additional band appeared at 37 kDa. Since the α-occludin H-279 antibodies are directed against the C-terminus of the protein, we proposed that the C-terminus of occludin is cleaved off upon infection. As control, probing of the blot with the above mentioned monoclonal α-occludin antibody did not detect the cleaved 37 kDa fragment, because the recognized epitope lies in the N-terminus of the protein (data not shown). Remarkably, this cleavage was dependent on HtrA, as the 37 kDa product was predominantly visible when cells had been infected with wild-type *C. jejuni* compared to the Δ*htrA* deletion mutant (Fig. 7a).

### In vitro cleavage assay and identification of a HtrA cleavage site in occludin

To confirm that the 37 kDa product of occludin is indeed cleaved by HtrA, we performed in vitro cleavage assays using recombinant proteins. For this purpose, N-terminally GST-tagged human occludin (monomers of ~ 100 kDa and dimers of ~ 200 kDa) was incubated with purified HtrA. After 16 h of incubation, the in vitro cleavage reactions were subjected to immunoblotting using α-occludin (H-279) and α-GST antibodies (Fig. 7b). The blot showed the appearance of the same sized C-terminal 37 kDa fragment of occludin through cleavage by HtrA. The remaining 63 kDa N-terminal fragment was also visible after staining with α-GST (Fig. 7b). Mapping of these cleavage products suggests that HtrA can cleave occludin in the second extracellular loop of the protein (Fig. 7c, top). Inspection of the occludin sequence in the second extracellular loop (amino acids 196–243) indeed identified the presence of a protease cleavage site corresponding to A/TGLYVD (Fig. 7c, bottom), which shares significant resemblance to the signature motif [VITA]-[VITA]-x-x-D-[DN] defined previously for preferential *H. pylori* HtrA cleavage sites in E-cadherin [50]. We also predicted the secondary structure of the loop to be in close vicinity of the cleavage site. This analysis indicates the presence of short elements of secondary structure flanking the cleavage site, whereas the site itself is located in a random coil region suggesting a high accessibility for cleavage by HtrA (Fig. 7d).

## Discussion

The tight junction complex is a key regulator of epithelial cell functions. Besides providing a selective barrier to the lumen, tight junction proteins have been shown to be involved in differentiation, cell-to-cell adhesion and proliferation of healthy epithelial cells [51]. In addition, tight junctions are an important target for microbial pathogens that manipulate the host, in order to support their own survival, spread and sometimes persistence. Out of a myriad of potential molecular targets, some bacterial and viral pathogens have selected a subset of proteins in the apical-junctional complex of epithelial cells [52]. For instance, *Clostridium perfringens* produces virulence factors that directly interact with tight junction proteins [26]. Eichner and co-workers [53] demonstrated that *C.* *perfringens* enterotoxin is able to destroy the epithelial cells, for which the toxin initially binds to tight junction proteins, using claudin-4 as a receptor. Another example is *Vibrio cholerae*, which produces a metalloprotease, haemagglutinin/protease (HA/P), which functions as a cytotoxin. In fact, HA/P degrades occludin, thereby affecting ZO-1. Since ZO-1 is bound directly to occludin, these events can induce a signaling pathway influencing the host cell actin cytoskeleton [54]. However, the cleavage site of HA/P in occludin has not yet been identified. More information is available for secreted HtrA produced by the gastric pathogen *Helicobacter pylori* that cleaves E-cadherin at various positions, and the cleavage sites have been recently identified by mass spectrometry and Edman degradation, respectively [50].

The most important result of the currently presented work is that HtrA of *C. jejuni* can cleave occludin in vitro, during infection of cultured polarised Caco-2 cells and in biopsies of campylobacteriosis cases in vivo, which affects its distribution in epithelial cells and various bacteria-host cell interactions. Recently, Elmi and co-workers [38] demonstrated in in vitro experiments that purified OMVs of *C. jejuni* are able to trigger the cleavage of E-cadherin and occludin, however, whether this cleavage is caused by a bacterial protease or activated human protease remained unclear. Here, we provide evidence that *C. jejuni* HtrA cleaves occludin directly. Our previous analysis of the cleavage sites in E-cadherin employed by *H. pylori* HtrA revealed that they are flanked by hydrophobic amino acids, preferably valine, isoleucine, threonine and alanine [50]. A consensus cleavage sequence has been defined to occur at the [VITA]-[VITA]-x-x-D-[DN] motif [50]. However, not all HtrA cleavage sites strictly confer this pattern and there is a certain degree of variability concerning the conservation and spacing of the C-terminal acidic residue [50]. To take this observation into account, we defined the more fuzzy pattern [VITA]-[VITA]-x(2,4)-[DN] for the identification of a HtrA cleavage site in occludin. The fact that only one site in the second extracellular loop matches this pattern and that this site is characterized by lack of secondary structure, renders it a perfect candidate for recognition and cleavage by HtrA. Interestingly, the size of the obtained 37 kDa C-terminal cleavage product is identical to that produced by *H. pylori* HtrA [55], which is associated with the relocation of occludin from tight junctions into the cytoplasm seen in another study [56], suggesting that both HtrAs may use the same cleavage site in occludin, which was identified here. However, a faint band of 37 kDa was also present in cell lysates upon infection with *C. jejuni* Δ*htrA.* We propose that it could derive from cleavage by another yet unknown secreted *C. jejuni* protease or by a host cell protease that maybe activated upon infection. One such candidate is Meprin-A, a metalloprotease, which is highly expressed at the luminal interface of the intestine and can cleave a variety of substrates in vitro, including occludin [57]. Another candidate is metalloprotease-9 (MMP-9). It was shown that activation and cellular export of MMP-9 led to the cleavage of occludin during infection by hepatitis C virus [58]. If Meprin-A and/or MMP-9 can be activated by *C. jejuni* is not clear and should be investigated in future studies.

Occludin contains two extracellular loops, which are involved in tight junction protein interactions and are important to maintain the cellular barrier function. In mice, it has been shown that inactivation of the occludin gene had only a slight effect on the barrier function of tight junctions for ions, as characterized by impedance spectroscopy, and thus did not result in pronounced gut leakage [59, 60]. The cell shape or growth behaviour of the epithelial knockout cells remained unchanged, and tight junctions were still being formed, suggesting that occludin may not essential for tight junction assembly. However, it is debated whether in a knockout animal other tight junction proteins can eventually functionally compensate the lack of occludin, at least in part [59, 60]. Nevertheless, instant effects on occludin cleavage by *Campylobacter* HtrA might cause a (temporary) barrier defect. Also, occludin deficiency has been discussed in the context of an increased macromolecule passage through epithelia, especially since occludin does influence the distribution of tricellulin (occludin presence drives tricellulin into the tricellular tight junction), a tight junction protein with important tightening function for the tricellular tight junction, at a site in the epithelium, where three or four cells meet [61].

These observations are in line with our experiments using Caco-2 cells and corresponding occludin knockout cells produced by CRISPR/Cas9 technology, showing that occludin is not absolutely essential for the formation of tight junctions. Our results suggest that there are downstream effects of occludin inactivation, as we clearly observed a decrease in presence of membrane-associated ZO-1. The polarization capacity of the knockout cell lines was followed over time by TER determination. This showed that inactivation of occludin resulted in slightly lower TER values compared to wild-type Caco-2 cells. From this, we conclude that polarization of the cells might have been decreased to some degree, and although tight junctions were still being formed, their function seems to be slightly impaired when occludin is inactivated. It appears that these cells are able to partly compensate the lack of occludin by restructuring other tight junction proteins, which may explain why levels of ZO-1 were slightly lower in our Caco-2 occludin knockout cells [36].

The C-terminal cytoplasmic tail of occludin comprises a coiled-coil domain, which is able to trigger homodimerization of the protein and binding to ZO-1 [62–64]. Previous work has also shown that disruption of epithelial barrier functions can be achieved by transfecting occludin mutant constructs lacking the intracellular tail [65], suggesting that the coiled-coil domain is functionally important for maintaining the cellular tight junction barrier [66]. Interestingly, immunoblotting of native and SDS-PAGE separated cell extracts revealed occludin dimers and monomers simultaneously on gels. The dimer is presumably an antiparallel intermolecular interaction by disulfide bond formation via cysteine residue 409 in human occludin [67]. It is concluded that the redox-dependent dimerization of occludin may play a regulatory role in the tight junction assembly under physiological and pathological conditions. We also have observed occludin dimers of the recombinant protein (Fig. 7b). Interestingly, we noted that HtrA cleavage terminated occludin’s dimerization properties. Thus, we propose that HtrA cleavage affects occludin functions by terminating its dimerization and interaction with ZO-1, to increase tight junctional permeability upon infection.

In line with our hypothesis, both adhesion and invasion of *C. jejuni* strains was increased in occludin knockout cells as compared to Caco-2 wild-type cells. Most likely, the tight junctions of the knockout cells did not provide full protection against the bacterial transmigration, so that more wild-type *C. jejuni* can reach the basal membranes, where they could adhere and invade. Our results suggest that loss of occludin in Caco-2 cells by CRISPR/Cas9 inactivation partially opened this barrier, which phenotypically mimics cleavage of the protein by HtrA, so that wild-type bacteria can better transmigrate between neighbouring cells. This provides a likely mechanism by which secretion of HtrA can assist *C. jejuni* in reaching the basolateral side of the epithelium and invade deeper tissues. In fact, using the adhesion proteins CadF or FlpA, *C. jejuni* typically invades epithelial cells through the fibronectin-based focal adhesion complexes at the basolateral membrane, which can only be targeted when the bacteria first penetrate the tight junctions of a confluent cell monolayer [68]. In line with this assumption, the ∆*htrA* mutant adhered to and invaded into occludin knockout cells at much better rates compared to wild-type Caco-2 cells.

**Fig. 8:**
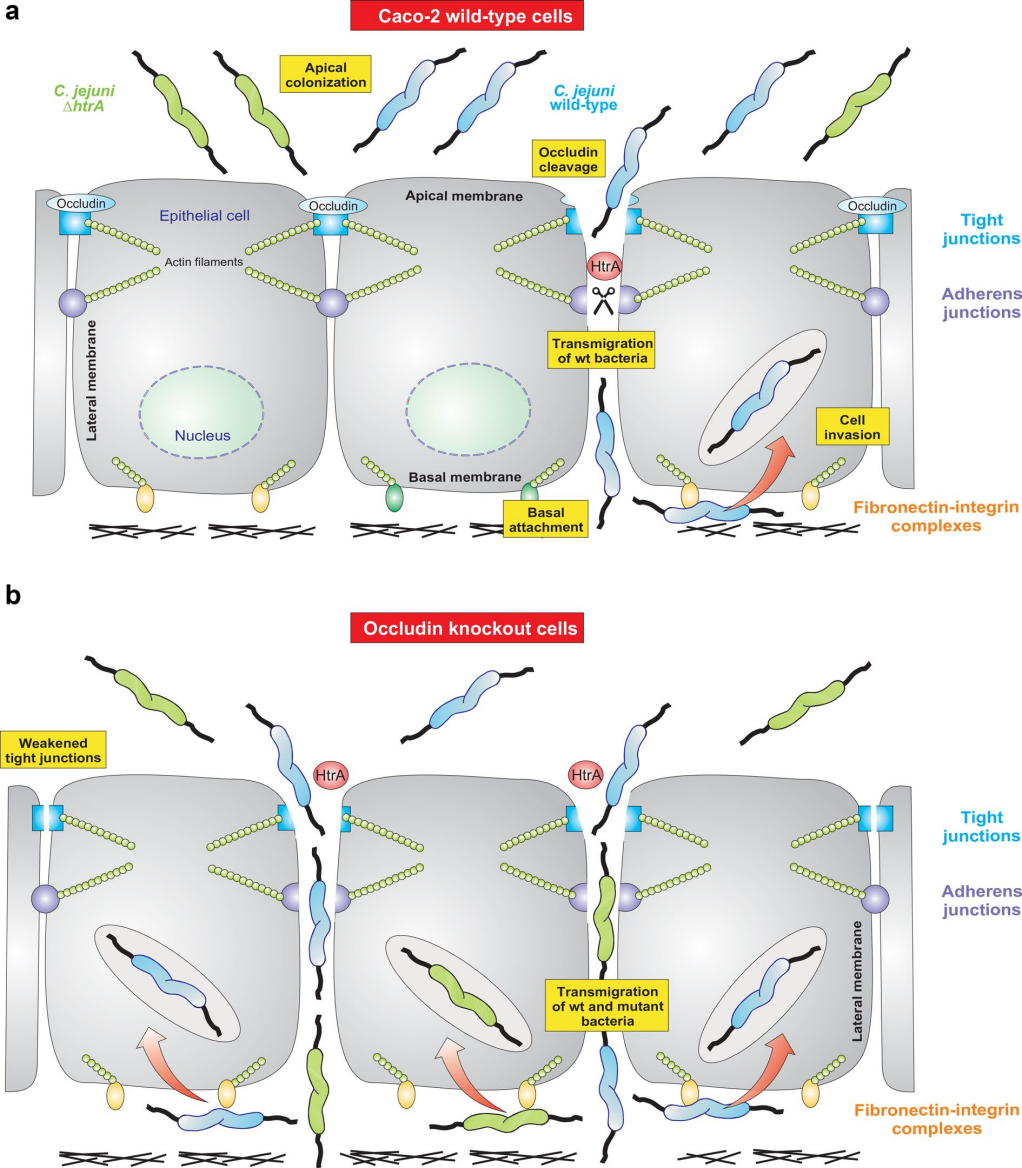
Model for the transmigration, adhesion and invasion by *C. jejuni* during infection of polarized Caco-2 cells depends on the expression of HtrA and tight junction protein occludin. **a** Upon infection of Caco-2 wild-type cells, *C. jejuni* exploits the secreted serine protease HtrA to cleave the apical tight junction protein occludin (this work) and the adherens junction protein E-cadherin [11]. Cleavage of both factors weakens the tight and adherens junctions, respectively, followed by entering of wild-type *C. jejuni* into the intercellular space between neighboring cells of the gut epithelium and paracellular transmigration. This enables the bacteria to reach basal surfaces and the fibronectin-integrin complex that connect the cells with underlying tissue. This complex is used as the receptor for the *C. jejuni* adhesins CadF/FlpA to bind and enter the epithelial cells from the bottom. As cleavage of the junctional proteins by HtrA is required for this outcome, ∆*htrA* mutant bacteria are strongly diminished in transmigration, adhesion and invasion of polarized Caco-2 cells. **b** During infection of Caco-2 cells with occludin knockout, the tight junctions are slightly weaker compared to wild-type cells as confirmed by TER measurement and immunofluorescence microscopy. This leads to enhanced transmigration, adhesion and invasion rates by wild-type *C. jejuni*. In addition, absence of occludin even allows *∆htrA* bacteria to transmigrate to some extent, resulting in increased cell binding and invasion, albeit still at lower rates compared to wild-type *C. jejuni*

Taken together, our working hypothesis is that *C. jejuni* aims to reach the basal side of polarized epithelial cells by transmigration through the tight and adherens junctions that are at least temporarily opened by the action of HtrA (Fig. 8). Cleavage of occludin may be just one option to open the cell-to-cell junctions. However, occludin is probably not the only target which *C. jejuni* uses to enable transmigration, because HtrA can also cleave the adherens junction protein E-cadherin [11]. In this way, *C. jejuni* impairs intestinal barrier functions, invades epithelial cells and achieves access to deeper tissues and even other organs. This is an intriguing new mechanism to breach the epithelial barrier of the human host and cause disease. More studies are clearly necessary to investigate this infection strategy and the role of secreted HtrA in more detail.
